# Refining the Canadian Assessment of Physical Literacy based on theory and factor analyses

**DOI:** 10.1186/s12889-018-5899-2

**Published:** 2018-10-02

**Authors:** Katie E. Gunnell, Patricia E. Longmuir, Joel D. Barnes, Kevin Belanger, Mark S. Tremblay

**Affiliations:** 1 Healthy Active Living and Obesity Research Group, Children’s Hospital of Eastern Ontario Research Institute, Ottawa, ON K1H 8L1 Canada; 2 Department of Psychology, Carleton University, 550 Loeb Building, 1125 Colonel By Drive, Ottawa, ON K1S 5B6 Canada

**Keywords:** Physical literacy, Motivation, Physical competence, Knowledge, Physical activity, Children, Factor analysis, Validity

## Abstract

**Background:**

The Canadian Assessment of Physical Literacy (CAPL) is a 25-indicator assessment tool comprising four domains of physical literacy: (1) Physical Competence, (2) Daily Behaviour, (3) Motivation and Confidence, and (4) Knowledge and Understanding. The purpose of this study was to re-examine the factor structure of CAPL scores and the relative weight of each domain for an overall physical literacy factor. Our goal was to maximize content representation, and reduce construct irrelevant variance and participant burden, to inform the development of CAPL-2 (a revised, shorter, and theoretically stronger version of CAPL).

**Methods:**

Canadian children (*n* = 10,034; M_age_ = 10.6, SD = 1.2; 50.1% girls) completed CAPL testing at one time point. Confirmatory factor analysis was used.

**Results:**

Based on weak factor loadings (λs < 0.32) and conceptual alignment, we removed body mass index, waist circumference, sit-and-reach flexibility, and grip strength as indicators of Physical Competence. Based on the factor loading (λ < 0.35) and conceptual alignment, we removed screen time as an indicator of Daily Behaviour. To reduce redundancy, we removed children’s activity compared to other children as an indicator of Motivation and Confidence. Based on low factor loadings (λs < 0.35) and conceptual alignment, we removed knowledge of screen time guidelines, what it means to be healthy, how to improve fitness, activity preferences, and physical activity safety gear indicators from the Knowledge and Understanding domain. The final refined CAPL model was comprised of 14 indicators, and the four-factor correlated model fit the data well (*r* ranged from 0.08 to 0.76), albeit with an unexpected cross-loading from Daily Behaviour to knowledge of physical activity guidelines (mean- and variance-adjusted weighted least square [WLSMV] χ^2^_(70)_ = 1221.29, *p* < 0.001, Comparative Fit Index [CFI] = 0.947, root mean square error of approximation [RMSEA] = 0.041[0.039, 0.043]). Finally, our higher-order model with Physical Literacy as a factor with indicators of Physical Competence (λ = 0.68), Daily Behaviour (λ = 0.91), Motivation and Confidence (λ = 0.80), and Knowledge and Understanding (λ = 0.21) fit the data well.

**Conclusions:**

The scores from the revised and much shorter 14-indicator model of CAPL can be used to assess the four correlated domains of physical literacy and/or a higher-order aggregate physical literacy factor. The results of this investigation will inform the development of CAPL-2.

## Background

Over the past decade, researchers, practitioners, and teachers have become interested in the concept of physical literacy given its relevance to healthy active living, physical education curricula, policy, public health, sport, and active recreation [1–3]. Although global consensus across researchers and practitioners on the definition of physical literacy has yet to be reached [4], in 2015 several Canadian organizations collectively adopted and recognized the definition set forth by the International Physical Literacy Association [5–7]. In Canada’s Physical Literacy Consensus Statement (Canadian Consensus Statement) [5], physical literacy is defined as the “motivation, confidence, physical competence, knowledge and understanding to value and take responsibility for engagement in physical activities for life” [5].

Alongside the rapid proliferation of interest and research on physical literacy grew the need to develop an assessment tool that could be used to derive valid and reliable physical literacy scores [2, 3, 8]. Although several instruments have been created to measure physical literacy [2], the Canadian Assessment of Physical Literacy (CAPL) is the only assessment of children’s physical literacy that has undergone extensive peer-reviewed and published validation efforts, including assessments of feasibility, validity, and reliability [8, 9]. The purpose of this study was to update the validity evidence for CAPL scores, with the goal of reducing participant burden while also maximizing validity evidence based on factor structure and content representation aligned with recent advances in physical literacy research and theory.

### Canadian assessment of physical literacy (CAPL)

The CAPL was developed to meet the demand for an assessment tool that could be used to produce valid and reliable scores that were representative of children’s progress on their physical literacy journey [9]. The creation of the CAPL involved consultation with practitioners (e.g., physical education teachers) and researchers, an extensive review of Canadian school physical education curricula, the identification of existing assessments, and the creation of novel assessments when no others existed (see [10] for additional details). The feasibility of the CAPL was examined through an iterative process to ensure that children could perform the assessment protocols, the time to administer the protocols was reasonable, and the personnel needed to implement the protocols was appropriate [10].

Currently, the CAPL is comprised of 25 indicators chosen to align with the internationally accepted and recently published Canadian Consensus Statement definition of physical literacy [5, 6]. Nonetheless, CAPL creators acknowledge competing definitions of physical literacy [4]. Despite the various definitions of physical literacy, it is important to note that the CAPL is anchored to the Canadian Consensus Statement definition promoted by sector leaders [5, 11] to include four essential and interrelated elements proposed by other researchers [2]: motivation and confidence (affective); engagement in physical activities for life (behavioural); knowledge and understanding (cognitive); and physical competence (physical). Each indicator can be used alone or in combination, depending on whether the goal of the assessment is an overall measure of physical literacy or a more focused evaluation of one domain or aspect. The four interrelated domains of the CAPL for children aged 8–12 years are: (1) Physical Competence; (2) Daily Behaviour; (3) Motivation and Confidence; and (4) Knowledge and Understanding.

With recognition that validation is an ongoing process [12], the CAPL has undergone extensive modifications to reflect advances in physical literacy theory and to account for results from investigations of score reliability and validity. Although many indicators of the CAPL domains have remained consistent across time, some indicators of each domain have been modified, removed, or added based on scientific evidence and theoretical considerations. For instance, the curl-up and push-up protocols originally envisioned as part of the CAPL were removed and replaced with the static plank hold [13]. Additionally, the initial movement skill assessment was modified to clarify/simplify scoring and for use in smaller spaces [14]. Subsequently, investigation of the factor structure along with feedback from experts through a Delphi process was undertaken to further validate and weight the CAPL scores [8].

### Factor structure of CAPL and current limitations

**Fig. 1:**
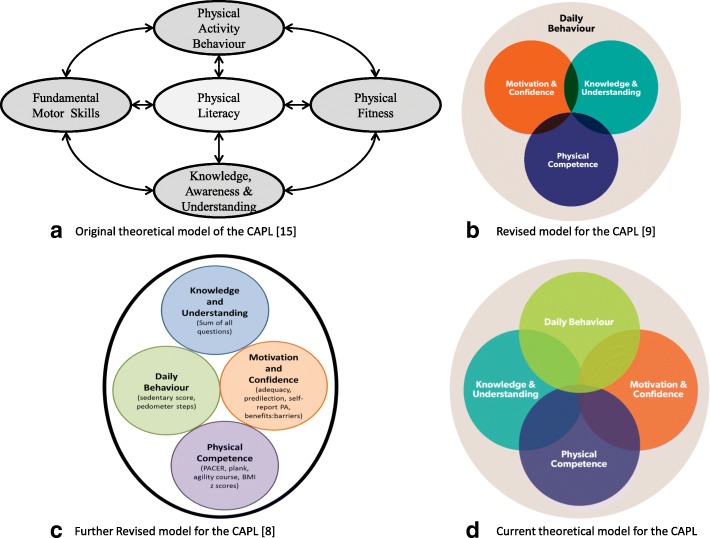
Historical and current configurations for the domains of the Canadian Assessment of Physical Literacy (CAPL).**a** Is adapted, by permission, from Pediatric Exercise Science. 2010;22(2):176–82. 10.1123/pes.22.2.176. © Human Kinetics, Inc. [15]. **b** Is reproduced, by permission, from Journal of Physical Activity and Health. 2016;13(2):214–22. 10.1123/jpah.2014-0597. [9]. **c** Is reproduced, by permission, from BMC Public Health. 2015;15:767 10.1186/s12889-015-2106-6. © Longmuir et al. [8]. BMI z: body mass index standardized for age and gender; CAPL: Canadian Assessment of Physical Literacy; PA: physical activity; PACER: Progressive Aerobic Cardiovascular Endurance Run

In its original conceptualization, the CAPL was comprised of four domains labelled (1) fundamental motor skills; (2) physical activity behaviour; (3) physical fitness; and (4) knowledge, awareness, and understanding (see Fig. 1a) [8, 15]. In an effort to determine the configuration and relative importance to physical literacy, a 3-stage Delphi process was undertaken wherein 19 experts in childhood physical activity and fitness evaluated the original CAPL model [8]. Results from the Delphi process indicated that the CAPL model should be reflected as three inter-related domains labelled (1) Physical Competence (subsuming measurements of motor skill and physical fitness); (2) Motivation and Confidence; and (3) Knowledge and Understanding, which together are encompassed by a fourth domain labelled (4) Daily Physical Activity Behaviour (see Fig. 1b).

At this juncture, it is important to acknowledge the debate within the physical literacy literature around the inclusion of physical fitness and physical activity as indicators of physical literacy [2, 4, 16]. Notably, indicators of physical fitness were included within CAPL given that Whitehead [16] has argued that physical literacy is a journey that requires the capacity to be physically competent. CAPL developers have argued that the measurement of fitness can be used to indicate an individual’s capacity to sustain physical activity for life [17]. Similarly, the creators of CAPL interpret the Canadian Consensus Statement definition to mean that when people value and take responsibility for engaging in physical activity, they will demonstrate this by being physically active. From this perspective, the creators of CAPL believe the definition of physical literacy includes physical activity behaviour as part of physical literacy. This perspective is consistent with the model proposed by Robinson and Randall [2] and the elements of physical literacy outlined in the Canadian Consensus Statement [5, 6]. Corroborating these arguments to include physical fitness and physical activity, the Delphi experts also recommended the inclusion of these two objectively assessed domains of physical literacy (i.e., Physical Competence and Daily Behaviour) and recommended that they receive more relative weight for creating an overall physical literacy score compared to the subjective domains (i.e., Motivation and Confidence, and Knowledge and Understanding). Finally, Francis and colleagues [8] recommended further inquiry into the factor structure of CAPL scores, with larger samples and factor analytic models to better demarcate the relative weighting of each CAPL domain in the overall composite score of physical literacy.

Following the recommendations from the Delphi process, data were collected with the intention of determining the relative importance of each CAPL domain and providing further validity evidence for the CAPL structure and scoring. To this end, Longmuir and colleagues [10] examined the factor structure of CAPL scores with a Canadian sample of 489 children. Results indicated that the CAPL scores should be reflected as four separate but inter-related domains of (1) Physical Competence, (2) Motivation and Confidence, (3) Knowledge and Understanding, and (4) Daily Behaviour (see Fig. 1c).

Despite the preliminary evidence, limitations of this past work were apparent. First, given the complexity of the full CAPL protocol and the modest sample size, not all indicators were included in the factor analytic model [10]. Second, rather than using each question from the Knowledge and Understanding questionnaire as indicators, one overall composite indicator was used. Finally, a careful inspection of the factor loadings obtained in the factor analysis called into question the salience of certain CAPL indicators for representing their respective domains. For example, standardized body mass index (BMI) z scores, total physical activity scores derived from pedometer step counts, and screen time scores were weak indicators (λs < 0.27) of their respective CAPL domains [10].

In the present study, using a much larger dataset, we re-examined validity evidence of scores from the full CAPL protocol based on factor structure and content representation alongside recent advances in the conceptualization of physical literacy, and relying on scientific and theoretical advances in physical literacy research. A second purpose was to reassess the relative weight of each domain for creating an overall physical literacy score. Our overall goal was to maximize content representation and validity evidence while reducing construct irrelevant variance and participant burden. The results from this study will set the stage for the development of CAPL-2 – a condensed and more theoretically aligned version of CAPL.

It is important to recognize that in this study we were not examining the validity of each indicator (e.g., grip strength) but, rather, examining how each indicator coalesces to demarcate a CAPL domain. Therefore, although we will present evidence for a reduced model of the CAPL, we do not wish to imply that the indicators removed were in some way invalid, but rather that they do not configure optimally, theoretically, statistically, or logistically in combination with the other indicators to create a particular CAPL domain. It is also important to note that initially we took a confirmatory approach to examine the *a priori* hypothesized model of CAPL based on past theory and empirical evidence [8, 9]. When the model did not fit the data, a more exploratory approach through the lens of confirmatory factor analysis was used; however, to avoid the negative consequences of data-driven specification searches [18], extensive discussion of substantive theory and alignment preceded data-driven modifications. Exploratory modifications were not made unless they were theoretically anchored and were supported by all co-authors. Further, all modifications were made with suggestions for cross-validation in future research.

## Methods

### Participants and procedures

A complete description of the participants and procedures for these data are presented by Tremblay and colleagues in this issue [19]. Briefly, Canadian children (*n* = 10,034; 50.1% girls) ranging in age from 8.0 to 12.9 (M_age_ = 10.6, SD = 1.2 years) completed CAPL testing. This project was approved by the Children’s Hospital of Eastern Ontario Research Ethics Board (Ottawa, Ontario; coordinating centre) and research ethics boards at each site. Parents or legal guardians provided written informed consent and children provided verbal assent.

### CAPL measures

The CAPL is comprised of standardized assessments with evidence of score reliability and validity in children aged 8 to 12 years [8, 14, 15, 20–25]. A detailed description of each assessment protocol can be found at www.capl-ecsfp.ca.

#### Physical competence

The Physical Competence domain was assessed with 7 indicators. (1) *Movement skills* were assessed with the Canadian Agility and Movement Skill Assessment (CAMSA; [15, 23]. The CAMSA can be used to assess children’s fundamental (e.g., sliding, catching, jumping, throwing, skipping, kicking, hopping), complex (e.g., acceleration and deceleration, rhythmic movement, hand-eye coordination), and combined (e.g., balance, coordination, equilibrium, precision, core stability) movement skills. Evaluators assessed the quality of skills performed and time taken to complete the skills. (2) *Muscular endurance* was assessed with the unlimited timed plank isometric hold in seconds [13]. (3) *Muscular strength* was assessed using a handgrip dynamometer in kilograms [24]. (4) *Flexibility* was assessed with the sit-and-reach protocol in centimetres [24]. (5) *Cardiorespiratory endurance* was assessed with the Progressive Aerobic Cardiovascular Endurance Run (PACER) shuttle run in number of laps completed [23]. Body composition was assessed via (6) *BMI z score* (objectively measured body mass divided by standing height squared [kg/m^2^], then standardized using age- and gender-specific BMI reference data and formulae based on the LMS method in accordance with the guidelines from the World Health Organization [25]) and (7) *waist circumference* in centimetres.

#### Daily behaviour

The Daily Behaviour domain was comprised of physical activity and screen time and assessed with 3 indicators. Physical activity was assessed directly with (1) *pedometer step counts* (initially with a Digi Walker pedometer [YAMAX Health & Sports, Inc., San Antonio, Texas]; since 2014 with a SC-StepRx pedometer [StepsCount, Deep River, ON]), which includes the number of steps taken every day [26]. The pedometer was worn on a waistband over the right hip and participants were required to have 4 valid wear days. (2) *Self-reported physical activity* was assessed by asking children to report the number of days during the past week (7 days) they engaged in at least 60 min of physical activity at a moderate to vigorous intensity. (3) *Screen time* was assessed via self-reported time spent on a school day and on a weekend day (a) watching television or (b) playing video games and/or using computers in recreational time with response options of 0 (none) to 6 (5 h or more). An overall screen time score was calculated as average daily screen time = [(hours of TV on weekdays × 5) + (hours of TV on weekend days × 2) + (hours of video games and computers on weekdays × 5) + (hours of video games and computers on weekend days × 2)]/7.

#### Motivation and confidence

The Motivation and Confidence domain was comprised of 5 self-reported indicators. A (1) *benefits to barriers* difference score (total perceived benefits minus total perceived barriers to physical activity) was calculated based on children rating their agreement on a scale of 1 (disagree a lot) to 5 (agree a lot) to items assessing physical activity barriers (10 items) and benefits (9 items) [20]. (2) *Adequacy* and (3) *predilection* subscale scores were used from 17 items taken from the Children’s Self-Perception of Adequacy in and Predilection for Physical Activity (CSAPPA) Scale [21]. Children responded to items using a structured alternative response format scored on a scale of 1 to 4. Finally, (4) *activity level compared to others* and (5) *skill level compared to others* were assessed with one item each in which children used a scale of 1 (a lot less active; others are better) to 10 (a lot more active; I’m a lot better).

#### Knowledge and understanding

The Knowledge and Understanding domain was comprised of 10 indicators [27]. Multiple items based on the Canadian provincial curricula for physical and health education for children in grades 4 to 6 were created to assess knowledge and understanding [27]. The content evaluated in the assessment included: knowledge of the (1) *Canadian Physical Activity Guidelines for Children and Youth* [28] and the (2) *Canadian Sedentary Behaviour Guidelines for Children and Youth* [29] (multiple-choice response options, scored as incorrect/correct); knowledge of the definition of (3) *cardiorespiratory fitness* and (4) *muscular strength* (multiple-choice response options, scored as incorrect/correct); knowledge of (5) *what it means to be healthy* (matching the word “health” to various descriptive phrases such as “being skinny”, “eating well”, “looking good”, “feeling good”, and “not being sick” when accurate; scored out of 6 for each incorrect/correct response); (6) c*omprehension and understanding* (fill in the blank with a word bank provided; scored out of 6 for each correct word in the appropriate blank space); knowledge of when to (7) *use safety equipment during various activities* (circle activities they do and check those that require safety gear; scored as correct/incorrect for each circled activity divided by the number of activities circled for a range of − 0.63 to 1); and knowledge on how to improve (8) *sport skills* and (9) *fitness* (multiple choice response options, scored as incorrect/correct). In its current form, the CAPL includes one indicator that was erroneously placed in the Knowledge and Understanding domain. The indicator (10) *preferred leisure time activity* (select preferred after-school activities from a range of active and inactive activities; scored as 0 [sedentary pursuits] and 1 [active pursuits]) was initially intended to serve as an indicator of motivation and confidence. For the current analyses, we will retain the indicator under Knowledge and Understanding to align with all previous CAPL publications and the current coding of CAPL domain scores.

### Data analysis

#### Preliminary analyses

Data cleaning procedures for outliers and data entry errors are outlined in Tremblay et al. [19]. All analyses were performed in Mplus 7.1 [30]. Although the total sample size was 10,034, not every child participated in each assessment. Therefore, sample sizes vary based on completion of assessment indicators. Missing data ranged from 1.7 to 6.4% on all variables except daily step count, which had 43.3% missing data. Given the high percentage of missing data on daily step count, we conducted tests of mean difference to examine possible differences between those who provided valid data and those who did not. Children with valid pedometer data were more likely to be girls (χ^2^_(1)_ = 109.85, *p* < 0.01), but no differences in age were found (*t* (9046.63) = − 1.26, *p* = 0.21).

#### Main analyses

Confirmatory factor analyses were performed in sequential order, beginning with the examination of the factor structure for each CAPL domain individually. Typically, confirmatory factor analyses are used in later phases of research to test *a priori* hypothesized models [18]. As such, confirmatory factor analyses were deemed appropriate because the CAPL has a strong theoretical and conceptual foundation with a clear factor structure supported by previous factor analyses and validation work [8, 9]. In all models, the metric of latent factors was set by constraining the variance to one. Error covariances were constrained to zero unless otherwise specified. Robust estimators that use all the available information to handle missing data were used. For Physical Competence, Daily Behaviour, and Motivation and Confidence, all indicators were specified as continuous and the robust maximum likelihood (MLR) estimator was used. For Knowledge and Understanding, 9 indicators were specified as categorical given their binary incorrect (0) or correct (1), or 0 to 6, scoring. The indicator representing the safety gear question was entered as continuous given that it could contain decimal or integer values. Because the Knowledge and Understanding domain included a combination of categorical and continuous indicators, the mean- and variance-adjusted weighted least square (WLSMV) estimator was used.

Each model was evaluated and modified using a combination of theory, statistical criteria (described below), and group consensus amongst all authors until an acceptable model was retained. For the Motivation and Confidence domain, a supplemental model was examined to determine if the indicator of preferred leisure activities fit under the domain of Motivation and Confidence (see “CAPL measures” section for justification of this supplemental analysis). Next, the final models from each individual domain’s confirmatory factor analysis were combined to examine a four-factor correlated measurement model using the WLSMV estimator attributable to the combination of continuous and categorical data. Finally, a higher-order model was tested to examine if physical literacy loaded onto each of the four domains that were measured by their respective indicators, and to determine the relative magnitude of each loading to inform the configuration of CAPL domains. To ensure missing data were not affecting the results, we conducted a sensitivity analysis to examine if this higher-order model yielded a similar pattern of results when using only participants who had complete data compared to including participants with incomplete data.

#### Evaluating model fit

Model fit for each factor analysis was examined alongside theoretical considerations and statistical guidelines, group consensus, and, in some cases, feedback received from CAPL experiences. A combination of goodness-of-fit statistics was used to determine statistical data-model fit, while recognizing that *p*-values were likely to be of limited use given the large sample size. Namely, although MLR or WLSMV chi-square statistics are presented for each model, they were not used to guide the evaluation of fit given their sensitivity to sample size [18]. In contrast, alternative indices were considered such as values close to or above 0.90 and 0.95 for Comparative Fit index (CFI) and values close to or below 0.08 and 0.06 for the root mean square error of approximation (RMSEA) [19, 31]. The 90% confidence interval around the RMSEA was also presented. Chi-square values were provided but not interpreted, as they are known to be affected by large sample sizes [18]. Additionally, we inspected all parameter estimates for out-of-range values (e.g., negative residual variances, correlations or standardized coefficients larger than 1). Modification indices were examined when the fit of the *a priori* specified model was poor. Modifications informed by statistical criteria alone were pursued only if all authors agreed that the modification made conceptual sense (e.g., adding an error covariance between two very similar indicators was justified given that they tap almost the same construct). Finally, the magnitude of factor loadings was examined to determine the contribution of each indicator to the hypothesized latent factor. Although researchers typically use statistical criteria to remove indicators (i.e., factor loadings below 0.30), we interpreted the magnitude of low factor loadings alongside theory and other model indicators to determine if an indicator should be retained, revised, or removed. For example, if a factor loading fell near the 0.30 value [18], we discussed whether or not that indicator was theoretically similar to other stronger indicators in the model. We also discussed whether or not it was conceptually a good indicator that enhanced content representation (or not) of the latent factor. Group consensus (i.e., 100% agreement among all authors) regarding removing, revising, or retaining an indicator had to be obtained before making any modifications.

## Results

### Physical competence

Model 1 of Physical Competence was a poor fit (MLRχ^2^_(14)_ = 7313.86, *p* < 0.001, CFI = 0.583, RMSEA = 0.231, 90% CI [0.226, 0.235]; see Table 1). Waist circumference had a problematic factor loading (λ = 0.99, *p* < 0.05). Because BMI z score and waist circumference are both indicators of body composition and are highly correlated (*r* = 0.80, *p* < 0.05), an error covariance between these two indicators was estimated in Model 2. The addition of the error covariance between BMI z score and waist circumference improved model fit, but model fit remained unacceptable (MLRχ^2^_(13)_ = 4670.81, *p* < 0.001, CFI = 0.734, RMSEA = 0.191, 90% CI [0.187, 0.196], *r*_BMIz.waist circumference_ = 0.781, *p* < .001). Grip strength, sit and reach, BMI z score and waist circumference had low factor loadings (λs < 0.32). Conceptually, BMI z score and waist circumference might not be good indicators of physical competence as they might load better on a body composition factor. Additionally, grip strength and sit and reach may be weak indicators of physical competence in comparison to the other indicators because they do not reflect the natural movements children engage in during active pursuits. Therefore, grip strength, sit and reach, BMI z score, and waist circumference were removed. Model 3 was estimated with only PACER, CAMSA, and plank as indicators. Model 3 was just identified and, therefore, fit indices could not be obtained. Nonetheless, inspection of the factor loadings indicated that the PACER, plank, and CAMSA were moderate to strong indicators of physical competence (λs > 0.55; see Table 1). Model 3 was retained as the final model for physical competence.

**Table 1:** Confirmatory factor analysis results of Physical Competence

	Model 1			Model 2			Model 3		
	λ	SE	R^2^	λ	SE	R^2^	λ	SE	R^2^
PACER	0.283*	0.012	0.080	0.799*	0.010	0.639	0.800*	0.011	0.640
Plank	0.273*	0.011*	0.075	0.564*	0.010	0.318	0.551*	0.010	0.303
CAMSA	0.094*	0.015	0.009	0.613*	0.010	0.375	0.602*	0.010	0.363
Grip strength	−0.420*	0.010	0.177	0.266*	0.014	0.071	–	–	–
Sit and reach	0.142*	0.010	0.020	0.161*	0.013	0.026	–	–	–
BMI z (reverse coded)	0.802*	0.012	0.643	0.318*	0.012	0.0101	–	–	–
Waist circumference (reverse coded)	0.999*	0.012	0.998	0.285*	0.012	0.081	–	–	–

*p < 0.01, λ = factor loadings, −- = item was not included in the model

Model 1 did not have any correlated errors between indicators, whereas Model 2 had a correlated error between BMI z and waist circumference

*BMI z* body mass index standardized for age and gender, *CAMSA* Canadian Agility and Movement Skill Assessment, *PACER* Progressive Aerobic Cardiovascular Endurance Run

### Daily behaviour

All pedometer step count scores were divided by 100 to reduce the variance of this indicator and assist with model convergence. Model 1 was just identified and therefore fit statistics could not be obtained. Inspection of the factor loadings indicated that self-reported screen time had the lowest factor loading (λ = 0.35; see Table 2). Although screen time and sedentary behaviours are conceptualized under the movement continuum [32], we reasoned that screen time might not belong conceptually in a measurement model of physical literacy given recent evidence showing that physical activity and sedentary behaviour are separate and weakly correlated movements [33, 34]. Therefore, a decision was made to remove self-report screen time from the model. Finally, although the factor loading of pedometer step counts was relatively weak (λ = 0.40), pedometers are considered to be a more direct indicator of physical activity compared to self-report physical activity [35]. As such, it was retained in the model on conceptual and content representation grounds. Model 2, removing self-report screen time from daily behaviour, could not be estimated with only two indicators. Therefore, the factor loadings of the Daily Behaviour domain were further examined in the full measurement model.

**Table 2:** Confirmatory factor analysis results of Daily Behaviour

	Model 1			Model 2		
	λ	SE	R^2^	λ	SE	R^2^
Pedometers step counts	0.396*	0.027	0.157	N/A	N/A	N/A
Self-report physical activity	0.471*	0.030	0.222	N/A	N/A	N/A
Self-report screen time (reverse coded)	0.345*	0.032	0.119	–	–	–

*p < 0.01, λ = factor loadings, N/A = not applicable. -- = item was not included in the model

### Motivation and confidence

Model 1 of the Motivation and Confidence domain provided an unacceptable fit to the data (MLRχ^2^_(5)_ = 1529.61, *p* < 0.001, CFI = 0.876, RMSEA = 0.176, 90% CI [0.168, 0.183]; see Table 3). Modification indices (Modification Index = 1265.62) suggested that there was a large error covariance between the indicators of “activity compared to others” and “skill compared to others”. We reasoned that this error covariance could be attributable to the similar wording and response options of these questions. Model 2, with an error covariance between these two indicators, provided an excellent fit to the data (MLRχ^2^_(4)_ = 365.42, *p* < 0.001, CFI = 0.971, RMSEA = 0.096, 90% CI [0.087, 0.104] *r*_activity compared to others and skill compared to others_ = 0.42, *p* < 0.01; see Table 3). Despite excellent model fit, we removed “activity compared to others”, for two reasons. First, the two items with the error covariance were very similar and might therefore cause redundancy in the model (i.e., each is not adding unique construct relevant variance). Second, the indicator “activity compared to others” had the lowest factor loading compared to the indicator “skill compared to others”. The model was re-estimated excluding “activity compared to others.” Model 3 for Motivation and Confidence provided an excellent fit to the data (MLRχ^2^_(2)_ = 188.94, *p* < 0.001, CFI = 0.979, RMSEA = 0.097, 90% CI [0.086, 0.109]; see Table 3) and was retained as the final model.

**Table 3:** Confirmatory factor analysis results of Motivation and Confidence

	Model 1			Model 2			Model 3		
	λ	SE	R^2^	λ	SE	R^2^	λ	SE	R^2^
Adequacy	0.778*	0.008	0.606	0.842*	0.007	0.679	0.848*	0.007	0.719
Predilection	0.732*	0.009	0.536	0.777*	0.007	0.604	0.760*	0.007	0.577
Benefits to barriers difference score	0.513*	0.009	0.263	0.511*	0.010	0.261	0.494*	0.010	0.245
Skill compared to others	0.712*	0.009	0.507	0.618*	0.009	0.332	0.620*	0.008	0.384
Activity compared to others	0.680*	0.010	0.463	0.576*	0.010	0.382	–	–	–

*p < 0.01, λ = factor loadings, −- = item was not included in the model

Model 1 did not have any correlated errors between indicators, whereas Model 2 had a correlated error between skill compared to others and activity compared to others

At this point, a supplemental analysis was run to examine if specifying activity preferences as an indicator of Motivation and Confidence provided a good fit. In this analysis, Model 3 of motivation and confidence was re-estimated, including the categorical indicator of activity preferences and using the WLSMV estimator given the combination of categorical and continuous indicators. Compared to Model 3 of motivation and confidence, including the activity preference indicator degraded model fit (WLSMVχ^2^_(5)_ = 509.66, *p* < 0.001, CFI = 0.936, RMSEA = 0.101, 90% CI [0.094, 0.109]). Although the indicator of activity preferences loaded relatively strongly on motivation and confidence (λ = 0.576, *p* < 0.01), a decision was made to not include this indicator because it decreased overall model fit of the domain and because it is a dichotomous indicator that may not provide as much content representation compared to the current indicators of motivation and confidence.

### Knowledge and understanding

Model 1 with all original indicators of Knowledge and Understanding provided an unacceptable model fit (WLSMVχ^2^_(35)_ = 627.92, *p* < 0.001, CFI = 0.881, RMSEA = 0.041, 90% CI [0.039, 0.044]; see Table 4). In Model 2, the indicators of safety, activity preferences, and screen time guidelines were removed because of their weak factor loadings (λs < 0.20; see Table 4). Model 2 provided an acceptable model fit (WLSMVχ^2^_(14)_ = 211.43, *p* < 0.001, CFI = 0.954, RMSEA = 0.038, 90% CI [0.033, 0.042]; however, select factor loadings remained weak (see Table 4). When considering content representation of each indicator to the domain of Knowledge and Understanding, and alongside weak factor loadings, we decided to remove the indicator “what it means to be healthy” given that it might be a better indicator of health literacy rather than physical literacy. Two additional indicators – “improve sport skill” and “improve fitness” – also had weak factor loadings. A decision was made to remove one of these indicators and retain the other to enhance construct representation. Although the indicator “improve fitness” had the higher factor loading, we removed it based on its conceptual content that might already be tapped through the cardiorespiratory fitness definition indicator. The indicator “improve sport skill” was retained because, despite its lower factor loading, it showed slightly better discrimination with only 50% getting the answer correct (compared to 80% getting the answer correct for “improve fitness”) and because no other indicators in the Knowledge and Understanding domain tapped into this piece of knowledge.

**Table 4:** Confirmatory factor analysis results of Knowledge and Understanding

	Model 1			Model 2			Model 3		
	λ	SE	R^2^	λ	SE	R^2^	λ	SE	R^2^
Cardiorespiratory fitness definition	0.582*	0.016	0.339	0.597*	0.016	0.357	0.626*	0.018	0.392
Muscular endurance definition	0.696*	0.016	0.485	0.719*	0.016	0.516	0.731*	0.018	0.534
What it means to be healthy	0.313*	0.014	0.098	0.305*	0.014	0.093	–	–	–
Physical activity comprehension	0.512*	0.013	0.262	0.510*	0.014	0.260	0.480*	0.015	0.231
Safety	0.158*	0.013	0.025	–	–	–	–	–	–
Improve sport skill	0.301*	0.016	0.091	0.299*	0.017	0.089	0.308*	0.017	0.095
Improve fitness	0.365*	0.018	0.133	0.348*	0.018	0.121	–	–	–
Activity preferences	0.195*	0.018	0.038	–	–	–	–	–	–
Physical activity guidelines	0.360*	0.016	0.130	0.331*	0.017	0.110	0.321*	0.017	0.103
Screen time guidelines	0.074*	0.021	0.006	–	–	–	–	–	–

*p < .01, λ = factor loadings, −- = item was not included in the model

Lastly, the indicator querying knowledge of the physical activity guidelines had a weak factor loading in Model 2. Interestingly, during the collection of CAPL data, administrators have noted that the response options for this question are too easy for children to guess because the highest response option is the correct response. In fact, 63% of children answered this question correctly, suggesting that the response options should be altered to provide better discrimination. Based on this informal observation, along with considerations of maximizing content representation for the Knowledge and Understanding domain in light of the number of indicators already removed, a decision was made to retain the physical activity guideline indicator given its conceptual relevance. Overall, decisions around retaining weak indicators in the Knowledge and Understanding domain (i.e., “physical activity guidelines” and “improve sport skills”) were made with an eye toward optimizing construct representation while recognizing that these items require refinement if they are to be included in CAPL-2. Model 3 provided a good fit to the data (WLSMVχ^2^_(5)_ = 97.39, *p* < 0.001, CFI = 0.970, RMSEA = 0.043, 90% CI [0.036, 0.051] and was retained as the final model.

### Four correlated domains of physical literacy

Each of the final models described above were placed into one model, allowing for correlations between all four domains. Model 1 was not a good fit (WLSMVχ^2^_(71)_ = 2887.684, *p* < 0.001, CFI = 0.871, RMSEA = 0.063, 90% CI [0.061, 0.065]; Table 5). There was a large cross-loading from the domain of Daily Behaviour onto the indicator of physical activity guidelines (Modification Index = 1619.77). Model 2 was estimated including this cross-loading and it provided a good fit (WLSMVχ^2^_(70)_ = 1221.29, *p* < 0.001, CFI = 0.947, RMSEA = 0.041, 90% CI [0.039, 0.043]; Table 5). The cross-loading of physical activity guidelines on Daily Behaviour was stronger (λ = 0.42) than on its hypothesized domain of Knowledge and Understanding (λ = 0.28). A decision was made to retain this cross-loading and the original target loading. The question asked children how long children in general should be active and, therefore, conceptually it pertains more to knowledge and understanding than their own actual behaviours. Other problems and possible solutions with this indicator are outlined in the discussion below. Correlations between each domain ranged from 0.08 to 0.76 (*p* < 0.01; see Table 6). The strongest correlation was between Daily Behaviour, and Motivation and Confidence (*r* = 0.76). The weakest correlation was between Knowledge and Understanding, and Motivation and Confidence (*r* = 0.08); however, this correlation was likely only significant because of the very large sample size. Therefore, caution is warranted when interpreting this correlation, as it is very weak and may not be practically meaningful.

**Table 5:** Confirmatory factor analysis results of four domains of CAPL

	Model 1			Model 2		
	λ	SE	R^2^	λ	SE	R^2^
Physical Competence						
PACER	0.776*	0.009	0.602	0.775*	0.009	0.601
Plank	0.515*	0.010	0.265	0.529*	0.010	0.279
CAMSA	0.651*	0.009	0.424	0.648*	0.009	0.420
Motivation And Confidence						
Adequacy	0.784*	0.006	0.615	0.777*	0.006	0.603
Predilection	0.790*	0.007	0.623	0.785*	0.007	0.616
Benefits to barriers difference score	0.536*	0.008	0.287	0.541*	0.008	0.293
Skill compared to others	0.611*	0.008	0.373	0.617*	0.008	0.381
Daily Behaviour						
Pedometer step counts	0.386*	0.017	0.149	0.402*	0.016	0.161
Self-report physical activity	0.543*	0.018	0.294	0.594*	0.014	0.353
Physical activity guidelines	–	–	–	0.415*	0.015	–
Knowledge and Understanding						
Cardiorespiratory fitness definition	0.460*	0.017	0.212	0.577*	0.018	0.333
Muscular endurance definition	0.537*	0.018	0.289	0.674*	0.018	0.455
Physical activity comprehension	0.499*	0.015	0.249	0.559*	0.016	0.313
Improve sport skill	0.299*	0.018	0.089	0.333*	0.018	0.111
Physical activity guidelines	0.634*	0.019	0.402	0.281*	0.018	0.275

*p < 0.01, λ = factor loadings, −- = item was not included in the model

*CAMSA* Canadian Agility and Movement Skill Assessment, *CAPL* Canadian Assessment of Physical Literacy, *PACER* Progressive Aerobic Cardiovascular Endurance Run

**Table 6:** Correlations between four domains of CAPL

	1	2	3	4
1. Physical Competence	–			
2. Daily Behaviour	0.519	–		
3. Motivation and Confidence	0.539	0.760	–	
4. Knowledge and Understanding	0.330	0.102	0.082	–

All correlations significant at *p* < .01

*CAPL* Canadian Assessment of Physical Literacy

### Higher-order model of physical literacy

**Fig. 2:**
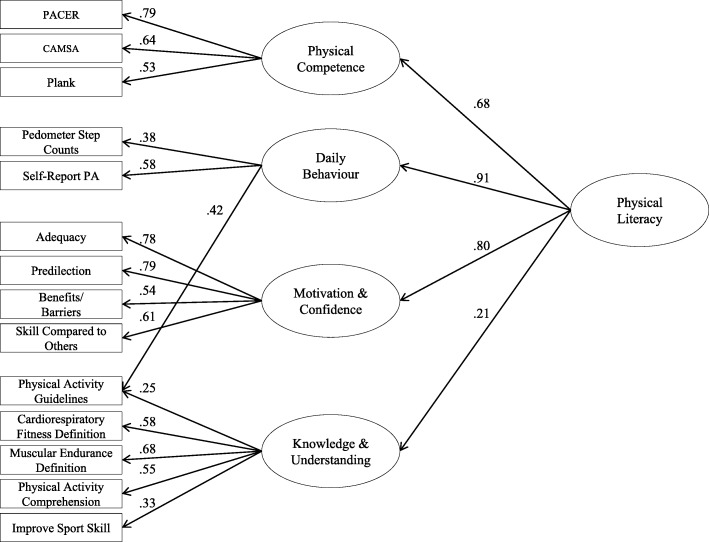
Higher-order confirmatory factor analysis results. All paths are statistically significant at *p* < 0.01. CAMSA: Canadian Agility and Movement Skill Assessment; PA: physical activity; PACER: Progressive Aerobic Cardiovascular Endurance Run

In the final model tested, we examined whether an overall physical literacy latent variable accounted for the correlations between the four domains. Using the final model from the four correlated domains model above, we found that the higher-order model had a good fit to the data (WLSMVχ^2^_(72)_ = 1827.18, *p* < 0.001, CFI = 0.919, RMSEA = 0.049, 90% CI [0.047, 0.051]; see Fig. 2). Daily Behaviour, Motivation and Confidence, and Physical Competence had the strongest factor loadings from physical literacy. Knowledge and Understanding had a significant, albeit weak, factor loading (see Fig. 2). Therefore, for the next version of CAPL, it is recommended that the domains be re-weighted such that Physical Competence, Daily Behaviour, and Motivation and Confidence have stronger weight (30 points each) than Knowledge and Understanding (10 points). The revised model suggested for CAPL-2, based on the good factor structure suggesting four intercorrelated domains of physical literacy, is shown in Fig. 1d.

An additional analysis using listwise deletion to examine the final higher-order model with only participants who had complete data (*n* = 5073) yielded a similar pattern of factor loadings on the pedometer step counts scores and self-report physical activity indicators (specific results from this supplemental analysis are available from the corresponding author upon request).

## Discussion

We examined the 25-indicator CAPL using factor analytic techniques to examine the validity of evidence based on factor structure and to determine the relative weighting of each domain of physical literacy. Using confirmatory factor analyses to test the *a priori* specified CAPL model and exploratory post-hoc modifications based on theory, group consensus, and statistical criteria, we found support for a revised and conceptually concise 14-indicator version of CAPL that maximized content representation while reducing construct irrelevant variance and participant burden. Aligning with the currently accepted Canadian Consensus Statement of physical literacy [5, 6], we found that a four-domain-correlated CAPL model of (1) Physical Competence, (2) Daily Behaviour, (3) Motivation and Confidence, And (4) Knowledge and Understanding provided a good fit; however, caution is warranted when interpreting the correlations between domains given the large sample size. Additionally, these four domains of CAPL could be subsumed under an overall physical literacy factor. Finally, based on the results from the higher-order confirmatory factor analysis, we suggest revising the CAPL weighting procedure for each domain such that three domains (Physical Competence, Daily Behaviour, and Motivation and Confidence) are equally weighted (i.e., 30 points each) whereas one domain (Knowledge and Understanding) has a weaker (i.e., 10 points) relative contribution to total physical literacy (see Fig. 1d).

### Refining CAPL

Four out of the seven original indicators comprising the domain of Physical Competence were removed because they did not load as strongly as the other indicators. In the first iterations of CAPL, BMI z scores and waist circumference were proposed to serve as indicators of “body composition”, subsumed under a domain labelled “physical fitness” [15]. As a result of the Delphi process, the CAPL model was revised and the domain of “Physical Fitness” was replaced with “Physical Competence”; yet, the indicators of BMI z score and waist circumference were retained under the Physical Competence domain, with a call for future research to assess their contribution. Our finding that the BMI z score was a weak indicator of Physical Competence corroborates previous findings [10], whereas the finding that waist circumference was a weak indicator was a novel finding. Conceptually, because BMI z score and waist circumference were originally proposed as indicators of “body composition” subsumed under “physical fitness”, we are not surprised that they represent weak indicators of the broader domain of Physical Competence. As such, we deemed the removal of these two measures appropriate and suggest that they may be better used as a separate outcome. Two additional indicators (i.e., sit and reach and grip strength) were removed given their weak factor loadings on Physical Competence. It could be argued that the CAMSA, PACER, and plank are better indicators of physical competence and, in turn, of physical literacy, because all tests have been shown to mimic realistic movements and durations of children’s experiences during physical activity. For example, the PACER mirrors the sporadic patterns of starting and stopping by children [23]; the CAMSA enables children to perform simple movements and complex skills in reaction to a changing environment [14]; and the plank assesses torso endurance, which is vital for effective and efficient use of the upper and lower extremities and spinal stability [13]. In contrast, the static flexibility and muscular strength assessments used herein may be less relevant to the physical competence of children of this age, who perform primarily dynamic rather than static movements. It is nonetheless possible that if a few alternative indicators of flexibility and muscle strength were also measured, there could have been additional latent variables that would better characterize these aspects of physical competence. Overall, our results confirm emerging evidence that the domain of Physical Competence pertaining to physical literacy is best characterized by indicators that capture whole body movement, including both skill and fitness components.

For conceptual reasons discussed in the Daily Behaviour domain’s results around the distinctiveness between physical activity and sedentary behaviours (including screen time), a decision was made to remove screen time as an indicator of physical literacy. Additionally, the Daily Behaviour domain is comprised of one subjective (i.e., self-report physical activity) and one direct (i.e., pedometer step counts) indicator. The subjective indicator had a stronger factor loading than that of the pedometer step counts. It is unlikely that this finding stems from the large amount of missing data since the results were similar when the model was rerun with only complete data; nevertheless, there were gender differences between boys and girls who provided valid pedometer data that could have impacted the results. Alternatively, the discrepant factor loadings could be related to what each indicator is actually measuring. In the past, researchers have found that self-reported physical activity and scores from more direct measures may be weakly correlated [36] and tap into somewhat different components of physical activity [35]. For example, children can self-report physical activities that direct measures of physical activity do not capture (such as swimming and cycling, which children report frequently) [35], complex upper body movements, and non-load-bearing activities [37]. Additionally, within the CAPL, the *number of days* in a *typical week* engaged in physical activity was assessed via self-report, whereas the *number of steps* taken over a *specific week* was assessed via pedometer. These discrepancies between measurements could be causing the differential magnitudes of the factor loadings. Nonetheless, both indicators served as moderately robust indicators of Daily Behaviour and were retained.

An unexpected finding from the Daily Behaviour domain was the large cross-loading from knowledge of physical activity guidelines onto daily behaviour in comparison to its hypothesized domain of Knowledge and Understanding. There are a few possible explanations for this finding. First, it is possible that the instructional stem for the self-report physical activity indicator (which asks the children to report how many days they have engaged in moderate and vigorous physical activity *for at least 60 min*) actually provided the answer to the subsequent question about how long children should engage in moderate to vigorous physical activity each day (i.e., the correct answer is 60 min). Second, as identified by CAPL administrators through informal feedback, the response option for the physical activity guideline question may have been too easy given that the correct response was the highest value listed. This response option is problematic because it is easy to guess and might have been cued by the self-report physical activity indicator, which includes “60 min” in the instructional stem. In other words, it is possible that the two questions are sharing variance given that children can link the responses of one to the stem of the other. Future research is needed to determine if altering the response options for the knowledge of physical activity guidelines can reduce this problematic cross-loading. Finally, it is worth noting that we did not remove knowledge of physical activity guidelines as an indicator of the Knowledge and Understanding domain and place it as an indicator of Daily Behaviour. Our decision to retain it as an indicator of knowledge and understanding and allow for the cross-loading was based on the conceptual content of the indicator. Because the indicator asks children to report how long kids *should* be active, we did not feel it was a good indicator of how long the children themselves were actually active.

Five out of 10 indicators from the Knowledge and Understanding domain were removed. Conceptually, many original indicators that have not undergone previous validation do not align with current physical literacy research. For example, knowledge of what it means to be healthy could be an indicator of health literacy rather than physical literacy. Removing self-report screen time from the Daily Behaviour domain and knowledge of screen time guidelines from the Knowledge and Understanding domain can be justified for similar reasons; they may not be conceptually linked to physical literacy given that sedentary behaviours are distinct from physical activity behaviours [33, 34]. Additionally, as noted in the “CAPL Measures” section, we identified an error in the CAPL manual such that an originally conceived indicator for motivation and confidence was erroneously placed within the Knowledge and Understanding domain (i.e., activity preferences). Not surprisingly, asking children their activity preferences was a weak indicator of their knowledge and understanding, and so was removed from that domain. Supplemental analyses indicated that the indicator did indeed load within the Motivation and Confidence domain, although it resulted in a slight deterioration in model fit. Given that the indicator is dichotomous and that the other indicators of Motivation and Confidence provided a superior model fit, combined with an eye toward reducing the participant burden of CAPL, we decided to retain the Motivation and Confidence domain without this indicator.

Knowledge about the safety gear required for physical activity may be a weak indicator of knowledge and understanding, given that the response format did not provide standardized response options. Indeed, this question had an unusual format where children first selected activities they personally engaged in and then, of those, they circled activities requiring safety gear. Consequently, the answers could vary widely.

The question around how to “improve fitness” was removed because a similar question (i.e., “improve sport skills”) was deemed to add more unique content representation whereas “improve fitness” may already be captured by the definition of cardiorespiratory fitness. We retained some indicators of Knowledge and Understanding that had weak factor loadings so as to retain content representation in light of the number of indicators removed. We believe that despite their weaker factor loadings, “improve sport skills” and “knowledge of physical activity guidelines” were querying unique information and that with further redevelopment of the remaining Knowledge and Understanding indicators, they may have stronger factor loadings. The results herein were used to inform the redevelopment of the knowledge and understanding indicators. Notably, the response options for the knowledge of physical activity guidelines indicator were changed, and another fill-in-the-blank question was added to the physical activity comprehension indicator (see Longmuir et al. [38] for further details).

Lastly, one indicator was removed from the Motivation and Confidence domain. “Activity compared to others” was removed to assist in reducing participant burden and reduce construct irrelevant variance attributable to similarity with another indicator. Otherwise, the Motivation and Confidence domain had strong indicators.

### Practical implications

The results of this study will inform the creation of CAPL-2 [38]. It is important to note that although we have eliminated indicators that had weak factor loadings on targeted CAPL domains (e.g., waist circumference, grip strength), these indicators in and of themselves may not be bad indicators of what they are purported to asses (e.g., grip strength and sit-and-reach scores can be valid indicators of muscle strength and flexibility, respectively [39, 40]). Therefore, researchers may wish to include these assessments depending on their research question. Additionally, when collecting longitudinal data with the CAPL, it would be prudent to use the same version of CAPL as previously administered such that change over time can be examined unambiguously. Lastly, as noted in the “Background” section, researchers around the world have debated whether or not physical activity should be included within the definition of physical literacy or whether it would be better conceptualized as an outcome of physical literacy. Within the CAPL, we believe that valuing and taking responsibility for engaging in physical activity situates physical activity as an inherent component of physical literacy. Nonetheless, a strength of the CAPL is that researchers are free to use (or not use) domains that are of interest to them, given their theoretical perspective. As such, it is possible to use the CAPL and omit the Daily Behaviour domain to assess physical literacy, as described in the CAPL Manual (available at www.capl-ecsfp.ca).

A key finding of this study, when taken in the context of past versions, conceptualizations, and operationalizations of the CAPL, is that physical literacy cannot be reduced only to fitness or motor skill assessments. Indeed, original conceptualizations placed strong emphasis on indicators of physical fitness; yet over the past decade, with emerging validation and physical literacy research, it has become apparent that physical competence encompasses more than fitness and body composition and that motivation and confidence are equally important for physical literacy.

### Strengths, limitations and future directions

A major strength of this study was the large sample size, which included children from 11 regions across Canada, enabling the assessment of all CAPL indicators. Notwithstanding these strengths, limitations of this study should be acknowledged. There was a large amount of missing data on the pedometer daily step count scores, and there were gender differences in the amount of missing data for pedometers. Although modern procedures were used to handle missing data, it is not known to what extent the missing data influenced the final conclusions. Nonetheless, sensitivity analysis of the final higher-order model without missing data (i.e., using listwise deletion *n* = 5073) revealed a similar pattern and magnitude of results to those we report herein. Second, although our decisions to remove or retain indicators were made based on statistical criteria, theory, and considerations of content representation, many of these decisions were ultimately subjective in nature. It is possible that alternative models would have fit the data well. Moreover, although we used confirmatory factor analyses, the post-hoc modifications were more exploratory in that they were based on discussion, consideration of theory, and statistical criteria. It is paramount that researchers continue to examine the factor structure of the CAPL found herein using new samples in different contexts. Researchers may wish to test alternative models that align with other definitions of physical literacy theory [4]. Additionally, researchers may wish to extend validity evidence based on internal structure by examining grade and gender invariance to determine if CAPL scores are being measured in the same way across genders and grade levels. Finally, the measures in the Knowledge and Understanding and the Motivation and Confidence domains are self-reported and therefore susceptible to social desirability or recall bias.

## Conclusions

Using confirmatory factor analyses, we found validity evidence for a shorter, more concise, and theoretically aligned CAPL. Scores from the final 14-indicator model of CAPL can be represented as four correlated domains of physical literacy representing Physical Competence, Daily Behaviour, Motivation and Confidence, and Knowledge and Understanding. Additionally, these four correlated domains can serve as indicators of overall physical literacy. Finally, based on the results, we advise that the relative weighting of each CAPL domain be revised such that Knowledge and Understanding receives lower relative importance (i.e., 10 points) for physical literacy whereas Physical Competence, Daily Behaviour and Motivation and Confidence receive equal weighting (i.e., 30 points each).

## Abbreviations

BMI: Body mass index
CAMSA: Canadian Agility and Movement Skill Assessment
CAPL: Canadian Assessment of Physical Literacy
CFI: Comparative Fit Index
CSAPPA: Children’s Self-Perception of Adequacy in and Predilection for Physical Activity Scale
MLR: Robust maximum likelihood
PACER: Progressive Aerobic Cardiovascular Endurance Run
RMSEA: Root mean square error of approximation
WLSMV: Mean- and variance-adjusted weighted least square
